# Genetic differentiation between and within Northern Native American language groups: an argument for the expansion of the Native American CODIS database

**DOI:** 10.1080/20961790.2021.1963088

**Published:** 2021-09-20

**Authors:** Jessica A. Weise, Jillian Ng, Robert F. Oldt, Joy Viray, Kelly L. McCulloh, David Glenn Smith, Sreetharan Kanthaswamy

**Affiliations:** a Forensic Science Graduate Program, University of California, Davis, CA, USA; b Molecular Anthropology Laboratory, Department of Anthropology, University of California, Davis, CA, USA; c School of Mathematical and Natural Sciences, Arizona State University, Glendale, AZ, USA; d Sacramento County District Attorney’s Crime Laboratory, Sacramento, CA, USA; e California National Primate Research Center, University of California, Davis, CA, USA

**Keywords:** Forensic sciences, population genetics, Native Americans, North America, languages, short tandem repeats (STRs or microsatellites)

## Abstract

The National Research Council recommends that genetic differentiation among subgroups of ethnic samples be lower than 3% of the total genetic differentiation within the ethnic sample to be used for estimating reliable random match probabilities for forensic use. Native American samples in the United States’ Combined DNA Index System (CODIS) database represent four language families: Algonquian, Na-Dene, Eskimo-Aleut, and Salishan. However, a minimum of 27 Native American language families exists in the US, not including language isolates. Our goal was to ascertain whether genetic differences are correlated with language groupings and, if so, whether additional language families would provide a more accurate representation of current genetic diversity among tribal populations. The 21 short tandem repeat (STR) loci included in the Globalfiler® PCR Amplification Kit were used to characterize six indigenous language families, including three of the four represented in the CODIS database (i.e. Algonquian, Na-Dene, and Eskimo-Aleut), and two language isolates (Miwok and Seri) using major population genetic diversity metrics such as F statistics and Bayesian clustering analysis of genotype frequencies. Most of the genetic variation (97%) was found to be within language families instead of among them (3%). In contrast, when only the three of the four language families represented in both the CODIS database and the present study were considered, 4% of the genetic variation occurred among the language groups. Bayesian clustering resulted in a maximum posterior probability indicating three genetically distinct groups among the eight language families and isolates: (1) Eskimo, (2) Seri, and (3) all other language groups and isolates, thus confirming genetic subdivision among subgroups of the CODIS Native American database. This genetic structure indicates the need for an increased number of Native American populations based on language affiliation in the CODIS database as well as more robust sample sets for those language families.

Supplemental data for this article is available online at https://doi.org/10.1080/20961790.2021.1963088 .

## Introduction

The Combined DNA Index System (CODIS) refers to the Federal Bureau of Investigation’s (FBI) software associated with forensic DNA databases maintained at the local, state, and federal levels. CODIS has three primary databases: the offender/arrestee, missing persons, and forensic casework databases. The offender reference database consists of profiles belonging to convicted individuals and arrestees depending on local and federal laws. When dealing with an offender profile, there are two primary ways in which identification is made: (1) the profile of an unknown offender is searched against the CODIS offender/arrestee database to either identify a suspect from known offenders or make connections between crimes and (2) generating a random match probability, the probability that a random individual from a given population exhibits the particular combination of alleles exhibited by a known, suspected individual. Because some alleles and allele combinations have a higher frequency in some populations than in others, separate databases for the different ethnic populations found in the US are required to account for population-specific allele frequencies when estimating random match probabilities [1]. Obtaining the random match probability for a suspected individual profile against each of the different ethnic databases provides a more complete and accurate result than that for the combined database. The National Research Council recommends that genetic differentiation among subgroups (i.e. genetic subdivision) of such databases be lower than 3% of the total genetic variation within that database to provide reliable random match probabilities.

Language affiliation has often been used to define membership in genetically distinct populations. While as many as 7 097 distinct languages are currently spoken worldwide, only 23 are spoken by more than half the world’s populations (www.ethnologue.com, accessed 13 May 2018). As a language changes over time, its divergence can be characteri­zed much the same way as gene divergence is illustrated using a phylogenetic tree [2–4]. While genes and languages may appear to exhibit parallel histories if they have dispersed together as populations expressing them diversify, their histories may lack congruence due to differences in genetic and linguistic histories or uncertainties in the language classification [5]. Geographic isolation and drift could drive local genetic and language differentiation; however, while geographic isolation of a popu­lation typically decreases its genetic diversity, evidence-based phonemic data suggest that such isolation may not necessarily lead to the erosion of language diversity [6]. Parental or ancestral languages produce daughter languages over time through cultural and language evolution. Language evolution can occur in several different ways, most notably through geographic separation and isolation, population growth and expansion, as well as language shift or the usurpation of one language by another more prestigious one as different populations interact [4]. Language families constitute languages that originate from the same parental or ancestral language [7]. For this study, a language family refers to the parental language from which several daughter languages and/or dialects derive. Additionally, a language isolate has two accepted definitions: (1) a language with no identifiable relationship with any other known language, appearing as an anomalous outlier, and (2) a language belonging to a family of which no other member languages survive [8].

While a minimum of 56 language families and isolates are represented among Native Americans in the continental US, only four are currently represented in the Native American CODIS database, i.e. the Algonquian, Eskimo-Aleut, Na-Dene, and Salishan language families. In the present study, a minimum of 25 samples representing each of six language families (including three of the four, albeit not necessarily the same member languages, represented in the CODIS database) and two language isolates were analyzed using the 21 autosomal loci included in the Globalfiler• PCR Amplification kit to characterize their genetic structure, composition and differentiation. That level of differentiation, represented by *F*_*ST*,_ was compared with values of 0.03 that are recommended by the National Research Council for generating reliable random match probabilities for Native American tribal samples [1].

Mitochondrial DNA (mtDNA) and Y chromosome-linked short tandem repeat (Y-STR) evidence reveals a greater genetic diversification among (albeit less diversity within) Native American populations speaking different languages than among populations speaking different languages in other parts of the world [9–12]. There are currently 29 language families and 27 language isolates widely recognized in North America, of which six language families (Algonquian, Na-Dene, Eskimo-Aleut, Iroquoian, Uto-Aztecan, and Yuman) and two language isolates (Miwok and Seri) were included in this study. The “farming language dispersal hypo­thesis” postulates that languages of the Uto-Aztecan language family differentiated *via* the spread of farming practices, but not necessarily genes, from Mexico into the American Southwest [13]. This is supported by a significant correlation between language families and genetic dispersal in which 90.7% of mtDNA haplotypes within a language family are population-specific while fewer than 10% are shared between or among populations [14]. However, notable mtDNA differences among some geographically separated daughter languages of the Uto-Aztecan language family suggest the possibility of significant levels of genetic variation within language families possibly due to adoption of the more prestigious Uto-Aztecan language by genetically different popu­lations that also adopted the new farming practices [15].

Native American populations have become further subdivided linguistically due to physical isolation [5] as they dispersed into the American continent at different times *via* the Beringian Strait beginning approximately 20–25 kya [16]. The isolation from Asia created a severe genetic bottleneck exacerbated by the Beringian Standstill (also known as the Beringian Incubation), lasting several thousands of years, and subsequent dispersals throughout the American continent [16] resulted in only five extant mtDNA haplogroups [12] (Table 1). Language and other cultural boundaries have created highly effective barriers to gene flow, even among populations sharing relatively small geographic areas of residence [17]. This isolation is reflected by geographically restricted alleles such as the albumin variants Naskapi and Mexico and the B2a sub-haplogroup defined by the mtDNA mutation 16483A [18].

**Table 1.** Tribal and sample distributions among the eight language families/isolates and the primary mtDNA haplogroups associated with each tribe represented in the sample set (*N*=502).

Language family/isolate	Tribe	Primary mtDNA haplogroup	Number of individuals	Total
Algonquian	Kickapoo	A	1	25
	Potawatomi	A	1	
	Cheyenne/ Arapaho	A and C	2	
	Chippewa	A	21	
Na-Dene	Apache	A	88	96
	Dogrib	A	6	
	Haida	A	2	
Eskimo-Aleut	Eskimo	A	44	44
Iroquoian	Cherokee	B, C, D	33	33
Uto-Aztecan	Cora	B	64	112
	Nahua-Atocpan	A and B	8	
	Huichol	B	30	
	Nahua- Cuetzalan	A	3	
	Tarahumara	A and C	7	
Yuman	Kiliwa	B	14	130
	Pai Pai	B	13	
	Cochimi	B and C	25	
	Cucupa	B	11	
	Hualapai	B and C	2	
	Kumiai	B	15	
	Yavapai	B	50	
Miwok (Language isolate)	–	B and D	33	33
Seri (Language isolate)	–	C	29	29

While numerous studies have been conducted on the correlation between language and mitochondrial genetic differentiation among Native American popu­lations [3, 16, 19–21], mtDNA, like Y-STRs, provides a limited perspective on genetic differentiation within and among populations due to its exclusively uni-parental (i.e. maternal) inheritance and the consequent absence of genetic recombination [12, 15, 22]. Conversely, nuclear genetic markers are inherited from both parents and undergo recombination producing a higher effective population size than mtDNA, thereby reducing the effects of genetic drift [23]. Furthermore, the use of several different nuclear loci allows the comparison of gene flow estimates that are not confounded by the inherent differences between maternally and biparentally inherited genes. As such, nuclear markers provide a more comprehensive understanding of genetic structure and composition within and among Native American language families and isolates.

Several studies focused on nuclear genetic differentiation among Native American populations have reported a nuclear genetic bottleneck congruent with mitochondrial evidence [5, 24–26]. Additionally, nuclear data have detected a greater inter-population genetic differentiation among Native American populations than that observed among other North American populations, including Caucasians, African-Americans, Asian-Americans, and Hispanics [1]. More recent studies reflect the presence of even greater levels of genetic variability among tribal populations than those based on CODIS-STRs [1], likely due to greater tribal representation and a more comprehensive geographic sampling strategy than in the former studies [27, 28].

The present study aims to determine the extent to which language differentiation among six Native American language families and two language isolates in North America is correlated with their nuclear genetic differentiation and whether or not the genetic composition of the current Native American CODIS database is truly representative of that of Native Americans in the US. This study used 21 autosomal STR loci in the Globalfiler• PCR Amplification Kit (Thermo Fisher Scientific, Waltham, MA, USA) to evaluate nuclear genetic diversity, differentiation, and structure within and among samples of six Native American language families and two language isolates that represent a wide geographic range covering the Arctic, the Midwestern, Southwestern, and Southeastern US, California/Great Basin and Mexico, including Baja California (Table 1), as previously done [27, 28]. While other studies have used many more STR markers [5, 29], the Globalfiler• panel consists of the most utilized human STR markers in the world for individual differentiation and identification; therefore, results based on these markers not only have significant value in forensics and human gene­tics research but also provide a better comparative basis for evaluating the genetic structure and composition of human populations around the world.

## Materials and methods

**Figure 1.**
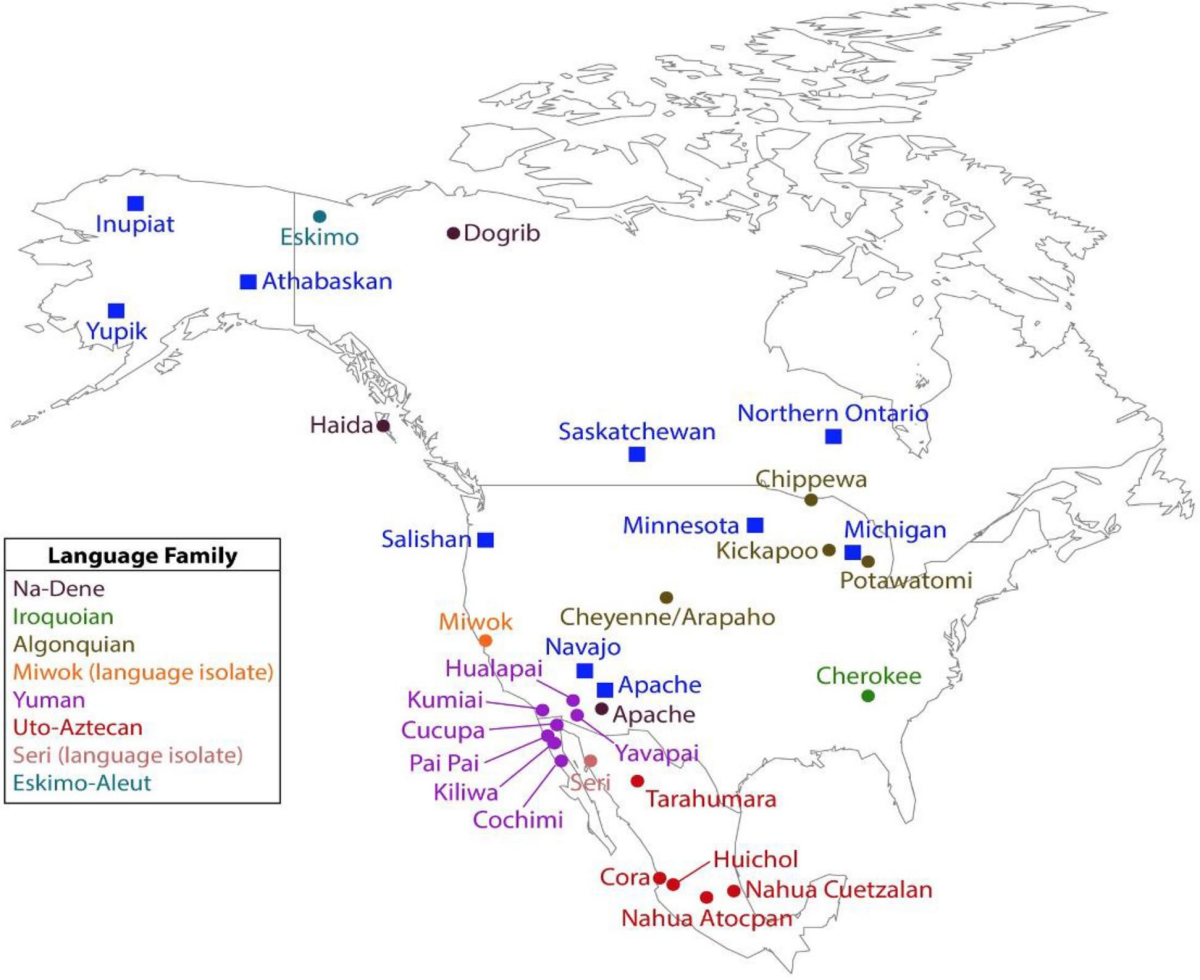
Map of tribes represented in Combined DNA Index System (CODIS) (solid blue squares) and the present study (solid circles). Note that all language groups included in the CODIS database, with the single exception of Salishan, are also represented in the present study. The legend identifies the language families of tribes included in this study.

Five hundred and two samples representing members of six language families and two language isolates (*n* ≥ 25 samples per language family or isolate; Table 1; Figure 1) met the minimum optimal concentration for amplification (0.1 ng/µL) and were subsequently genotyped for this study. These samples were selected post-quantification from approximately 1 935 DNA samples that were collected and extracted from whole blood using conventional techniques for various genetic studies [12, 14, 15, 18, 21, 24, 30, 31] and an additional 152 samples extracted from blood, buffy coat, serum, and plasma using the QIAamp DNA Blood Mini Kit (QIAGEN, Redwood City, CA, USA) following manufacturer protocols as described in previous studies [27, 28]. DNA quantities from the combined 2 087 samples were determined in duplicate on an Applied Biosystems 7500 Fast Real-Time PCR System (Applied Biosystems, Foster City, CA, USA) using the Quantifiler• Duo DNA Quantification Kit (Thermo Fisher Scientific) following manufacturer protocols. The low proportion of samples quantified at above 0.1 ng/µL DNA is likely due to their age and variable storage conditions.

The concentrations of the 502 extracted DNA samples, as well as the Standard Reference Material (SRM) 2391c reference DNA (National Institute of Standards and Technology, Gaithersburg, MD, USA), were normalized to approximately 1.0 ng/µL and amplified using the Globalfiler• PCR Amplification Kit following manufacturer protocols. The Globalfiler• Kit consists of the following autosomal STR loci that were selected to maximize discrimination potential for human identification and kinship analyses [32]: CSF1PO, FGA, TH01, TPOX, vWA, D3S1358, D5S818, D7S820, D8S1179, D13S317, D16S539, D18S51, D21S11 D1S1656, D2S441, D2S1338, D10S1248, D12S391, D19S433, D22S1045, and SE33. The amplified samples were diluted in Hi-Di Formamide (Applied Biosystems) and run on a 3130*xl* Genetic Analyzer (Applied Biosystems) with POP-4 polymer (Applied Biosystems) according to run conditions and protocols recommended by the manufacturer. Profiles were analyzed using GeneMapper• ID-X v 1.4 (Applied Biosystems) and the Local Southern sizing method. Samples with saturated off-scale alleles were diluted based on the severity of the pull-up and re-injected.

The allele frequency distribution, average number of alleles (N_A_), observed heterozygosity (HO), and expected heterozygosity (HE) were estimated using ARLEQUIN v3.5.1.2 [33] to characterize language-specific allele distribution patterns and the extent of genetic diversification within and among the language families and isolates. To ensure that differing sample sizes did not bias allelic diversity, estimates of average allelic richness (N_R_) were calculated across all 21 loci for each language group and isolate after their sample sizes were normalized to the smallest population size using the Allelic Diversity Analyzer (ADZE) software programme [34]. ARLEQUIN was also used to calculate the *F*-statistics *F*_*IS*_ (local) and *FIT* (total) inbreeding coefficients, and *FST*, the proportion of genetic variance in a population that is due to differences among subdivisions within that language sample; pairwise *F*_*ST*_, the degree of differentiation between pairs of language samples, was also calculated to provide insight into the historical connections among these languages [35]. The statistical significance of the pairwise *F*_*ST*_ computations was determined with a probability distribution constructed from permutation tests (*N* = 1 000) with Bonferroni corrections for multiple comparisons. The *P*-values for deviations from Hardy-Weinberg Equilibrium (HWE) for each locus in each language group were estimated with GENEPOP 4.2 [36] and ARLEQUIN, respectively.

An analysis of molecular variance, AMOVA [33], was performed using ARLEQUIN based on all 21 STR loci to ascertain if the language affiliation of Native American populations contributed to the extant genetic structure. Statistical significance of the AMOVA values was estimated by a permutation test (10 000 permutations) at the 0.05 level of proba­bility. CONVERT v1.31 [37] was used to determine the number and frequency of private alleles present in each population sample. A model-based clustering method featured in the STRUCTURE v2.3.4 software programme [38] was used to infer genetic groups using the 21 STR loci genotype frequencies. All runs were based on the admixture model that assumes each person has ancestry in multiple genetic clusters. The model assumes there are *K* genetically distinct clusters (where the true value of *K* is typically unknown), each of which is characterized by a set of allele frequencies at each locus. Individuals in the sample were assigned probabilistically to a specific genetic group or jointly to two or more genetic groups if their genotypes indicate they are admixed. Each run used 500 000 estimation iterations for *K* = 1 to 8 after a 100 000 burn-in length. Each run was carried out five times for each value of *K.* The log probability of the data (Ln P(D)) and delta *K* (i.e. the *K* value with the highest Ln P(D) and lowest standard deviation) were used to determine the true number of genetically distinct populations [39] represented by the data.

## Results

N_A_ estimates for the eight language families or isolates ranged from 5.52 (Eskimo) to 9.43 (Uto-Aztecan). No deviation in N_A_ estimates attribu­table to variation in sample sizes of language groups was detected (Table 2). For instance, the Yuman (*n* = 130) and Algonquian (*n* = 25) language families had the largest and smallest sample sizes, respectively, but the N_A_ estimates from both these groups were comparable, i.e. 7.62 and 7.95, respectively. Moreover, the highest N_A_ estimate represented the Uto-Aztecan language family (9.43), only the second largest language sample in this study (*n* = 112), and the smallest N_A_ estimate represented the Eskimo-Aleut language family (5.52), whose sample size (*n* = 44) was almost twice that for the Algonquian language family (*n* = 25) which represented the smallest sample size. All language groups and isolates included in this study contained N_R_ values between 4.68 (Na-Dene) and 6.61 (Algonquian), reflecting a minimal impact of sample size (Table 2). The values of HO and HE in Table 2 also appeared to be uninfluenced by sample size with HO values ranging from 0.66 (Seri *n* = 29) to 0.78 (Algonquian *n* = 25 and Miwok *n* = 33) and EH values ranging from 0.64 (Seri *n* = 29) to 0.77 (Algonquian *n* = 25 and Iroquoian *n* = 33). All language groups exhibited either no marker or only a single marker violating HWE at the 0.01 level of probability with the exception of the Yuman, which had four loci not in HWE (Supplementary Tables S1–S8). None of the language groups were in HWE at the *P* = 0.01 level of probability (Table 2), and when all groups were combined, seven loci violated HWE (Supplementary Table S9).

**Table 2.** Allele number (N_A_) and observed (HO) and expected (HE) heterozygosity for each language.

Language family/isolate	N	N_A_	N_R_	HO	HE
Algonquian	25	7.95	6.61	0.78	0.77
Seri (isolate)	29	8.43	5.87	0.66	0.64
Iroquoian	33	6.30	5.03	0.74	0.77
Miwok (isolate)	33	8.33	6.57	0.78	0.76
Eskimo-Aleut	44	5.52	6.32	0.68	0.69
Na-Dene	96	8.90	4.68	0.72	0.74
Uto-Aztecan	112	9.43	5.90	0.70	0.73
Yuman	130	7.62	6.19	0.72	0.74

None of the language groups were in Hardy-Weinberg Equilibrium (HWE) at the *P* = 0.01 level. Note the absence of correlation between sample size (N) and any of the three diversity parameters, i.e. N_A_, HO, and HE, and between the number of alleles (N_A_) and either heterozygosity parameter.

Pairwise and population-specific *F*_*ST*_ estimates and average *F*_*IS*_ are presented in Table 3. The pairwise *F*_*ST*_ values ranged from 0.011 (between the Na-Dene and Yuman speakers) to 0.113 (between the Eskimo-Aleut and Seri speakers), and all were statistically significant at the 0.05 level of probability. Both the Eskimo-Aleut language family and the Seri language isolate exhibited the greatest average genetic divergence from the other populations with mean pairwise *F*_*ST*_ values of 0.069 and 0.072, respectively, while the Yuman language family had the lowest population-specific *F*_*ST*_ (0.028). *F*_*IS*_ values (Table 3) were highest for the Uto-Aztecan (0.039), followed by the Iroquoian language family (0.035). Miwok, Seri, and Algonquian language speakers exhibited a lack of inbreeding reflected by their negative *F*_*IS*_ values. Overall, *F*_*IS*_, *F*_*ST*_, and *F*_*IT*_ estimates across all language samples were 0.019, 0.032, and 0.051, respectively.

**Table 3.** Pairwise (below diagonal) and population-specific *F*_*ST*_, and *F*_*IS*_ estimates based on the 21 autosomal STR loci analyzed for the eight language families/isolates.

	Algonquian	Na-Dene	Eskimo-Aleut	Iroquoian	Seri (isolate)	Uto-Aztecan	Yuman	*F*_*ST*_	*F*_*IS*_
Algonquian	–							0.029	−0.019
Na-Dene	0.016	–						0.031	0.018
Eskimo-Aleut	0.045	0.059	–					0.069	0.017
Iroquoian	0.017	0.025	0.063	–				0.034	0.035
Seri (isolate)	0.064	0.057	0.113	0.077	–			0.072	−0.043
Uto-Aztecan	0.023	0.020	0.072	0.024	0.047	–		0.033	0.039
Yuman	0.017	0.011	0.054	0.019	0.057	0.015	–	0.028	0.026
Miwok (isolate)	0.024	0.028	0.076	0.012	0.087	0.027	0.024	0.040	−0.029

Overall *F*-statistics for all eight language samples: *FIS* = 0.019, *FST* = 0.032, and *FIT* = 0.051. Note that the average of the three pairwise values of *FST* among the three language families represented in the Native American CODIS database (Algonquian, Na-Dene, and Eskimo-Aleut) is 0.04, exceeding the 0.03 value recommended by the National Research Council for estimating random match probabilities.

**Figure 2.**
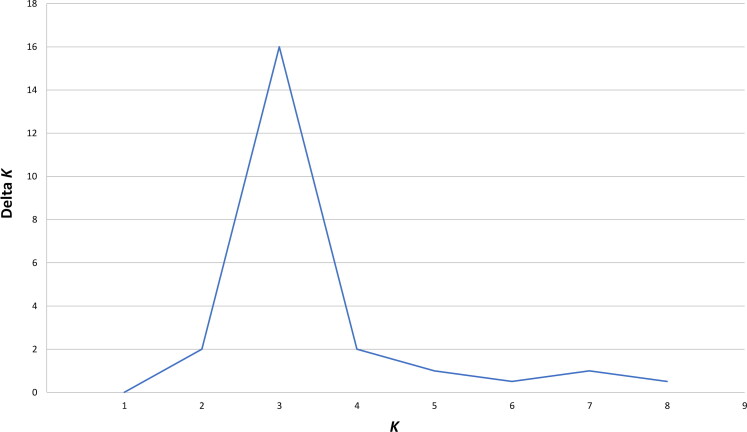
Delta *K* plot illustrating likelihood of all *K* values ranging from one to eight true genetic groups required to incorporate all language families and isolates in the dataset accurately; *K* = 3 was found to be the most probabilistic scenario.

**Figure 3.**
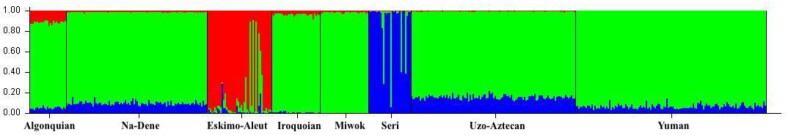
STRUCTURE plot comprising individuals from the six language groups and two language isolates, assuming the most probabilistic model of three true genetic groups (*K* = 3). The scale at the left margin reflects assignment probabilities to each of the three groups. The *x*-axis represents individuals from populations sorted according to their language groups/isolates. Each individual is represented by a vertical stacked column of colour-coded admixture proportions that reflect genetic contributions from each of the three true genetic groups.

**Figure 4.**
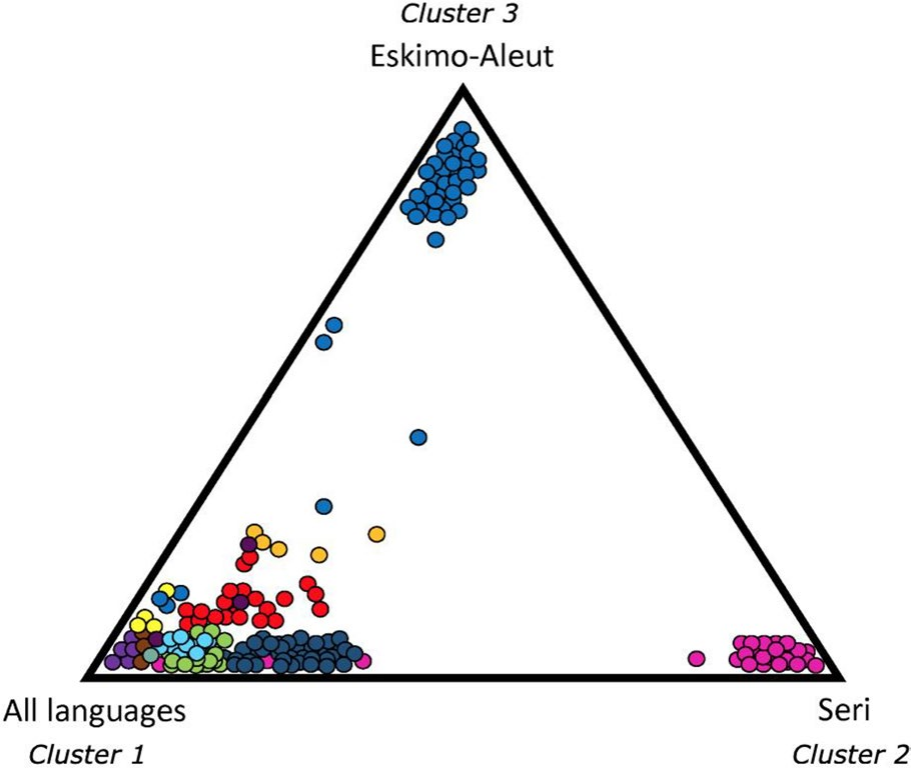
Language and genetic correlation among study individuals reveals three separate clusters: Clusters 2 and 3 consist exclusively of speakers of Seri and Eskimo-Aleut, respectively, while Cluster 1 comprises speakers of all other languages included in this study.

The AMOVA showed that genetic differences among individuals speaking different languages represent 4%–5% of the total genetic variation observed in this study, and these differences were statistically significant at the 0.05 level of probability. Based on the Ln P(D) and delta *K* results, there are three distinct genetic clusters (*K* = 3) indicating genetic subdivision among the eight language families or isolates (Figure 2). Assuming the most probabilistic model of three “true genetic groups” (*K* = 3), the STRUCTURE analysis reflected a strong correlation between genotypic distributions and membership in each of the three groups (Figure 3). While many of the individuals display partial membership in more than a single one of the three “true genetic groups”, suggesting high levels of genetic admixture, Eskimo-Aleut and Seri speakers were predominantly assigned to two different of the three clusters, represented by red and blue, respectively, in Figure 3, with members of all remaining six language families or isolates (Figure 4) being predominantly assigned to the third “true genetic cluster”, represented by green in Figure 3. Table 4 provides the average and ranges of assignment probabilities of all individuals included in this study to the predominant “true genetic group” representing their respective language families/isolates based on the STRUCTURE analysis (Figure 3). Only five Eskimo-Aleut and four Seri individuals exhibited average assignment probabilities lower than 50% (and one individual of each language sample exhibited a probability lower than 10%) to their respective “true genetic group” (Figure 3). Three additional Eskimo-Aleut samples and 13 Uto-Aztecan samples exhibited assignment probabilities between 60% and 70% to the predominant “true genetic group” representing their respective language groups (Figure 3). While the samples exhibiting assignment probabilities lower than 10% might represent misassigned ancestry, they were not removed from our analyses because it cannot be known for sure that this is the case. An alternative explanation for their lower average probabilities of assignment to their language group’s predominant “true genetic group” is unreported admixture with individuals belonging to the other unrelated language groups.

**Table 4.** The mean, range, and standard deviation (SD) of average assignment probabilities (averaged over five runs for each language family/isolate) of individuals to the predominant “true genetic group” of their language family or language isolate based on the STRUCTURE analysis illustrated in Figure 3.

Language sample	Mean	SD	Lowest value	Highest value
Algonquian	0.790	0.057	0.681	0.887
Na-Dene	0.851	0.034	0.738	0.910
Eskimo-Aleut	0.837	0.248	0.096	0.987
Iroquoian	0.948	0.018	0.884	0.969
Seri (isolate)	0.891	0.236	0.074	0.995
Uto-Aztecan	0.745	0.038	0.656	0.814
Yuman	0.854	0.025	0.799	0.903
Miwok (isolate)	0.983	0.010	0.970	0.989

Each individual was assigned probabilities of membership in one or more of the three “true genetic groups” identified by the Delta Plot in Figure 2. The Eskimo-Aleut and Seri language families exhibited the most variable assignment probabilities, probably due to misassigned or shared ancestry, either through common ancestral alleles or recent genetic admixture with individuals belonging to the other language groups.

## Discussion

This study aimed to ascertain if genetic diversity correlates with language group differentiation in Native American populations genotyped at the CODIS loci using the Globalfiler• PCR kit and, if so, whether samples representing Native Americans in the CODIS database are truly representative of Native Americans in the US. While classifications of Native American languages into language families by comparative linguists often differ, the composition and representativeness of language families employed in this study is recognized by more conservative comparative linguists [40]. The Algonquian, Na-Dene, Eskimo-Aleut and Salishan language families, which alone represent Native Americans in the CODIS database, display several unique alleles not found in most other North American groups, including the mtDNA 16192 variant [31] and the Albumin Naskapi allele AL*Naskapi [18], possibly as the result of admixture among the ancestors of these three northern language families whose dispersal patterns overlapped [41]. Sampling biases resulting from combining individuals from different distinct subpopulations into larger language samples for this study likely resulted in all the language groups not conforming to HWE conditions. *FST* and *FIS* values (Table 3) also point to varying levels of genetic drift and subdivision across language groups. The average of approximately eight alleles per locus observed in this study is consistent with previous descriptions of other population samples [1, 42]. Comparable estimates of HO and HE in the Alaskan tribes have been reported [1] with an average value of 0.70, while the Eskimo-Aleut samples in the present study exhibited mean values of 0.68 and 0.69, respectively. Subdividing samples by geography, without regard to language affiliation, has previously produced slightly higher estimates of HO and HE (0.72 and 0.73, respectively) [27].

The AMOVA shows that approximately 95% of genetic variation within the language groups is broadly concordant with other reports of worldwide interpopulation variation [43]. The lack of agreement in HO and HE estimates between language and geographic samples is consistent with the AMOVA results in this study, which failed to support the strict linguistic grouping. This may result from geographic contiguity among most of these groups, possibly favouring gene flow and genetic admixture among their speakers. Conversely, the STRUCTURE analyses revealed a lack of evidence of general clustering among six of the eight language affiliations, broadly attesting to the migration and subsequent assimilation of speakers of North American indigenous languages into earlier arriving immigrants. The existence of stratification based on STRUCTURE is due to the separation of most Eskimo-Aleut and Seri speakers from the other language samples, pointing to the pronounced influence of geographic distance or historical isolation on genetic and language differentiation as reported previously in the Seri [44]. This is in stark contrast to previous assessments [5] that suggested that the distribution of genetic variation among Native Americans reveals closer parallels between genetic and language patterns of differentiation; populations sampled from disparate and unevenly distributed geographic regions that represented more divergent major language reservoirs throughout North America (3 localities), Central America (8 localities), and South America (18 localities) would likely reinforce stronger correlations between language and genetic diversification. Integrating up to 678 STR loci may have provided a finer resolution of clinal variation among language samples, resulting from geographic isolation by distance [5].

The *F*-statistics obtained in this study across all individuals (*FIS*=0.019, *FST*=0.032, *FIT*=0.051) provide evidence to support that the loss of heterozygosity levels, which contributed to each group’s violation of HWE is caused by non-random mating, genetic subdivision, and genetic drift among the language samples. The use of these estimates to ascertain genetic structure and gene flow overcomes sampling issues because it considers resampling over replicate populations [35, 36]. This method of estimating *FST* performs well in cases with moderate amounts of gene flow and large population sizes as it does not tend to overestimate genetic subdivision [35, 45].

Language group-specific *FST* values indicate varying allele frequencies within language families/isolates and average allele frequencies across all language samples included in this study. The low-to-moderate pairwise *FST* values suggest that not all language families and isolates examined are differentiated, likely resulting from increased gene flow or recent common ancestry. Pairs of language groups that are geographic neighbors tend to be more genetically similar than groups that are more geographically distant, regardless of whether the languages are closely related [6]. In conjunction with the STRUCTURE analysis, the pairwise *FST* comparisons show that the Eskimo-Aleut and Seri language samples, which, incidentally, are fixed for mtDNA haplogroups A and C, respectively – comprise the most genetically distinct individuals in the study, likely due to their extant geographic isolation from the other samples. It has previously been shown that the Seri population experienced a genetic bottleneck due to reduced gene flow *via* historical isolation and is distinct from populations in the Uto-Aztecan language group [44] that live in close geographic proximity.

Ignoring group structure, an overall *FIT* of 0.051 points to a general deviation from HWE frequencies due to an excess of homozygotes even when all samples are combined. As *FIS* estimates indicate any deviation of the average allele frequency from HWE is probably due to consanguineous mating, the positive *FIS* values for Na-Dene, Eskimo-Aleut, Iroquoian, Uto-Aztecan, and Yuman speaking groups may reflect an increased relatedness among individuals who speak those languages rather than geographic isolation. In contrast, while inbreeding and geographic isolation are related phenomena, the Miwok and Seri language samples exhibit negative *FIS* values due to the absence of significant inbreeding despite being isolated linguistically or geographically. This finding suggests that these isolated language groups are more susceptible to genetic distinction due to loss of genetic variation from isolation and drift and not due to consanguineous mating.

Interestingly, while the overall level of genetic diversification among the language samples in this study revealed by the *FST* analyses (0.032) was very similar to earlier values reported (*FST*=0.03) [1], based on a small collection of tribal samples, it was somewhat lower than the 0.04 value previously reported [27] based on geographical samples and the same value for only those same three language groups (Algonquian, Na-Dene, and Eskimo-Aleut) representing Native Americans in both the CODIS database and our own study. The slightly lower degree (∼1%) of genetic differentiation in our study supports the notion that language groups are less differentiated than geographic groups because indigenous languages in North America spread more readily than the migration of people (i.e. gene flow) [27]. This is also in agreement with earlier comprehensive analyses that used large datasets to find that genetic and language distances between populations are less correlated with geographic distance in North America compared to most regions worldwide [6].

The geographic distribution of Native Americans has changed dramatically over the past five centuries owing to unique historical events such as early Spanish colonization, European immigration, and forced resettlement [46]. Migration events, isolation by distance, and cultural diffusion are among the factors that can contribute to variation in rates of genetic, geographic, and language differentiation. Because languages are not only inherited linearly but can diffuse laterally across geographic space [47], they change at a faster rate than genes resulting in discordances between genetic and language evolution over distances greater than 10 000 km [48]. It has been maintained that a broad association between language and genetic differentiation exists [20], but the lack of connections among language families and the absence of internal structures in them prevent languages and genes from evolving in synchrony [48]. Additionally, recent shared history in human populations can further reduce the correlation between language and genetic diversification [5, 6]. A weak association between genetic and language variation is particularly true of Native American populations, which have a relatively short genetic history in the Americas and have been very recently relocated to reservations or small geographic locations inhabited by speakers of multiple language families [49, 50].

The Apache and Navajo tribes in the CODIS database are southwestern US speakers of the Athabaskan languages of the Na-Dene language family [51, 52] and closely genetically resemble Athabaskans in Alaska and Canada. The Michigan, Minnesota, and northern Ontario tribes in the CODIS database all belong to the Algonquian language family and also closely resemble each other genetically, but only represent a minority of Native Americans in the US. As such, the current CODIS database comprises only members of the Algonquian (four languages), Na-Dene (the Athabascan, Navaho, and Apache languages), Eskimo-Aleut (the Inupiat and Yupic languages), and the Salishan language families while omitting the vast majority of tribes speaking other languages in North America and Mexico. This study’s genetic assessment of Native American language groups makes it evident that geographic barriers among distantly located populations result in both genetic and language isolation. As Hunley and Long [53] observed, there may be pressure to maintain a language at particular geographical locations due to the cultural complex to which it belongs; however, genes may be free to flow in these situations. As such, this study has highlighted the lack of genetic correlation among most language families and isolates.

When the *FST* value for the three language fami­lies shared between our dataset and the CODIS database (i.e. Algonquian, Na-Dene, and Eskimo-Aleut) are combined, their *FST* value is 0.04, 33% higher than the 0.03 *FST* value recommended by the National Research Council for random match probability generation [1]. When the five additional language families or isolates are combined with these three language families present in the CODIS database, *FST*=0.032, a value much closer to the recommended value, indicating the necessity to increase the number of language families included in the Native American CODIS database. The addition of a still greater number of language families to the CODIS database could undoubtedly provide a Native American database more representative of all Native Americans in the US and whose *FST* value is lower than the recommended value of 0.03. The use of frequency databases comprising additional language groups spoken by Native American profile contributors would provide more accurate random match probabilities, even if it does not account for the more rapid and complicated spread of languages than genes in North America between geographically disparate populations [53]. The CODIS database should be expanded to cover other geographically and linguistically heterogeneous groups of Native Americans to reflect the levels of extant genetic diversity among them more precisely so that more accurate match probability estimates can be computed with adjustments for population structure.
